# Cellular and molecular meniscal changes in the degenerative knee: a review

**DOI:** 10.1186/s40634-018-0126-8

**Published:** 2018-04-19

**Authors:** Mariano López-Franco, Enrique Gómez-Barrena

**Affiliations:** 1 Servicio de Cirugía Ortopédica y Traumatología, Hospital “Infanta Sofía”, Madrid, Spain; 2 Servicio de Cirugía Ortopédica y Traumatología, Hospital Sur de Alcorcón, Madrid, Spain; 3 Departamento de Medicina de la Universidad Europea de Madrid, Madrid, Spain; 4 Cirugía Ortopédica y Traumatología, Hospital Universitario La Paz, IdiPAZ, Universidad Autónoma de Madrid, Madrid, Spain

**Keywords:** Knee meniscus, Knee degeneration, Meniscal degeneration, Cellular and matrix changes

## Abstract

**Background:**

The important role of knee menisci to maintain adequate knee function is frequently impaired since early stages of knee joint degeneration. A better understanding of meniscal impairment may help the orthopaedic surgeon to orient the treatment of the degenerative knee. This review focuses on changes in meniscal cells and matrix when degeneration is in progress.

**Main body:**

Differences in the meniscal structure and metabolism have been investigated in the degenerative knee, both in experimental animal models and in surgical specimens. Cell population reduction, extracellular matrix disorganization, disturbances in collagen and non-collagen protein synthesis and/or expression have been found in menisci along with knee degeneration. These changes are considered disease-specific, different from those due to aging.

**Conclusion:**

Significant cellular and matrix differences are found in menisci during knee degeneration. These investigations may help to further progress in the understanding of knee degeneration and in the search of more biological treatments.

## Review

Osteoarthritis (OA) is a progressive disabling disease, resulting from the pathological imbalance of degradative and reparative processes in the different constituents of the joint. In the knee, menisci are assumed to deteriorate similarly to other joint elements. Knee menisci play an important role in the complex biomechanics of the knee joint. Meniscal injuries, partial or total meniscectomy, or meniscal degeneration, are considered to contribute to the development or progression of knee osteoarthritis (Fairbank, 1948; Gale et al., 1999; Lohmander et al., 2007; Song et al., 2008; Englund, 2009; Englund et al., 2009). In a normal knee joint, meniscal functions include (Radin et al., 1984; Fithian et al., 1990) load distribution, shock absorption, assistance with joint lubrication, and stability, particularly when the anterior cruciate ligament (ACL) is deficient. However, in spite of these paramount functions that may be altered during knee degeneration, the role of menisci in the onset and development of OA is not well understood.

The ability of menisci to perform mechanical functions is based on their cellular and biochemical composition and, perhaps more importantly, in the organization and interactions of their constituents. Macroscopic and microscopic changes have been observed in the meniscus along the degenerative process of the knee. This paper reviews the basic science behind meniscal changes during knee degeneration, comparing the available information about normal and degenerative joints. This will include microstructure and composition aspects oriented to cells and extracellular matrix constituents, particularly addressing collagen, proteoglycans, and other proteins such as COMP. Furthermore, various cellular and extracellular events occurring in knee degeneration will also be discussed, including cell proliferation, apoptosis and necrosis, but also calcification (Table 1).

**Table 1:** Meniscal differences between the normal and the degenerative knee

	MENISCUS: normal knee	MENISCUS: degenerative knee
Gross anatomy	Translucent, smooth and glistering	Dark yellow to brown or reddish color, roughened and fibrillated surfaces
Cell population	0.12% of the meniscus weight (around 98 cells per 0.1 mm^2^)	Decreased
Proliferating cells	Frequently observed	Almost absent
Necrosis and apoptosis	Almost absent	Frequently observed
Cells clusters	Exceptionally	Frequently observed
Calcification	No	Frequently observed
Collagen	Dense framework of collagen fibers. Type I (90%)	Reduced and disrupted or fragmented
Proteoglycans	< 1% and 80% sulfated	Initially increases. Tend to decrease in advances stages
COMP	High amounts. Same heterogeneous distribution than cells, decreasing from the red to the white zone	Decreased

## Normal meniscus

### Gross anatomy

**Fig. 1:**
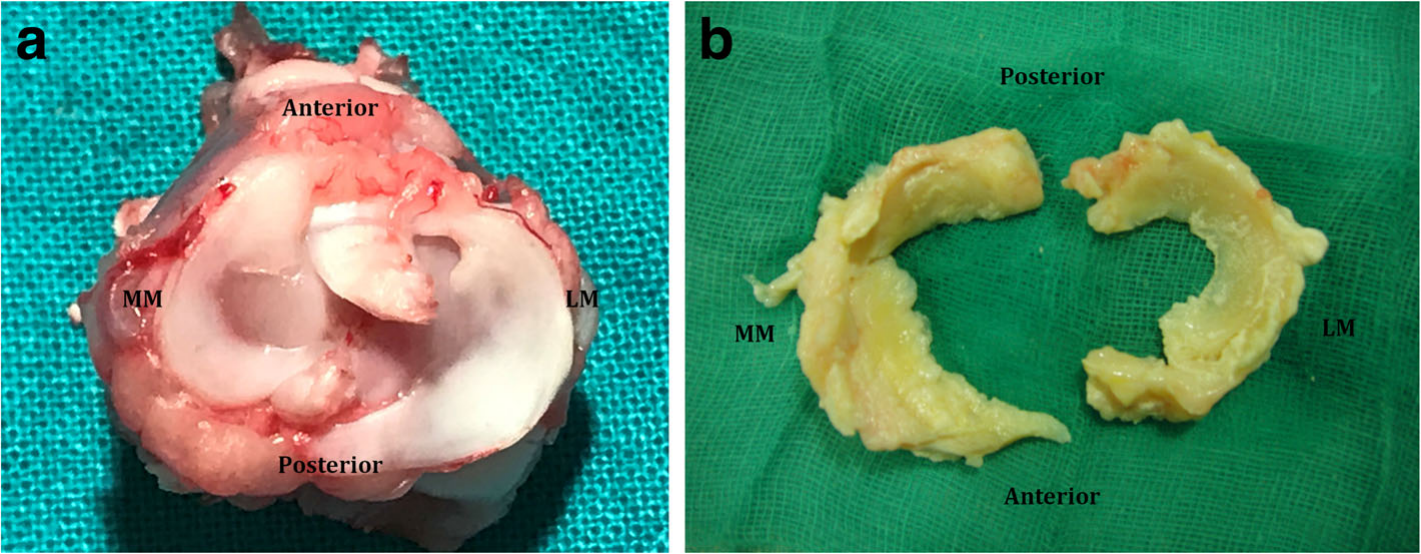
**a** Right knee joint from a healthy rabbit. **b** Human menisci retrieved from an osteoarthritic left knee. MM: medial meniscus; LM: lateral meniscus

The medial meniscus is C-shaped and the lateral is semicircular in shape. Both menisci have been classically divided in the anterior horn, the body and the posterior horn (Messner & Gao, 1998). Their surfaces have been described as translucent, smooth and glistering (Pauli et al., 2011). The meniscal horns are attached, via meniscal insertional ligaments, mainly to the tibial bone (Messner & Gao, 1998) (Fig. 1a).

### Microstructure and composition

Knee menisci contain a mixed population of cells, while the extracellular composition consists mainly of water and a type I collagen network (Fithian et al., 1990). Proteoglycans and other non-collagenous proteins play an important role stabilizing the extracellular meshwork. Therefore, these are crucial to maintain the structural integrity and mechanical properties of the menisci.

The outer 10% to 30% of the medial meniscus and the outer 10% to 25% of the lateral meniscus sustain vascularity (Arnoczky & Warren, 1982). Three zones can be distinguished in the menisci regarding vascularization: the outer peripheral or red zone (highly vascularized), the red-white zone (partly vascularized) and the inner or white zone (minimally or not vascularized). The red zone has a composition close to tendons, while the white zone is closer to hyaline cartilage. The anterior and posterior horns are more vascularized than their bodies (Arnoczky & Warren, 1982), more closely related to the red meniscal zone than to the white zone.

Normal human meniscal tissue is composed of 72% water, 22% collagen, 0.8% glycosaminoglycans (GAGs), and only 0.12% DNA, representing the cells (Herwig et al., 1984). Apparently, the water content of meniscal tissue is higher in meniscal tissue samples from the posterior horn than from the body or the anterior horn, but samples from superficial and deeper layers had similar water content (Proctor et al., 1989). On a dry weight basis, normal adult menisci contained 78% collagen, 8% non-collagenous proteins, and 1% hexosamine (Ingman et al., 1974). Hexosamines, particularly N-acetil-glucosamine, are determinant in the proteoglycan metabolism.

### Cells

A relatively small number of cells embedded in a dense extracellular matrix are responsible for the synthesis of this extracellular matrix. Historically, two types of morphologically distinct subpopulations, unrelated to the regionalization of the meniscus, have been identified. First, fusiform cells, located along the entire superficial margin of the meniscus, have been compared to flattened chondrocytes from the superficial zone of the articular cartilage. Second, cells lying interiorly to the superficial zone are described as oval or polygonal in shape and have been considered similar to the chondrocytes from the transitional and radial zones in the articular cartilage (Ghadially et al., 1983; Ghadially et al., 1978). These meniscal cells are detected in isolation, in pairs or in short rows, and are either randomly arranged or disposed in longitudinal rows between bundles of dense collagen fibers. Histological and biochemical studies indicate that the outer two-thirds of the meniscus are organized like fibrocartilage, whereas the inner one-third of the meniscus is arranged like hyaline cartilage.

More recently, four major morphologically distinct classes of cells were identified within the adult rabbit meniscus using antibodies to cytoskeletal protein (Hellio Le Graverand et al., 2001a). The first class corresponds to fusiform cells previously identified along the superficial margin of the tissue. Second and third classes are confined to the fibrocartilage-like region of the meniscus. Meniscal cells in each of these two classes are linked via gap junctions, but differ in the number of cell processes. The fourth class of meniscal cells is confined to the hyaline-like region and consists of round cells that display no direct contact.

### Collagen

The meniscal body consists predominantly of a dense framework of collagen fibers. Although several collagen types (I, II, III, V and VI) are present, type I (over 90%) is the most abundant (Shindo et al., 1981; Eyre & Wu, 1983; Cheung, 1987; McDevitt & Webber, 1990; Wildey et al., 2001; Hellio Le Graverand et al., 2001c).

The distribution of the different collagen types shows significant regional variations. The amount of collagen types I and III is considerably higher in the medial meniscus from skeletally mature rabbits (Hellio Le Graverand et al., 2001a). Except for trace amounts (< 1%) of collagen types III and V, the peripheral two-thirds of bovine menisci consist solely of collagen type I, whereas type II collagen (60%) predominated over type I collagen (40%) in the inner third (Cheung, 1987).

Throughout development and adulthood, differences in collagen concentrations can be observed: collagen types III and V are predominantly found pericellularly and within meniscal surface layers (Eyre & Wu, 1983; Bland & Ashhurst, 1996). A matrix containing collagen types I, III and V is found in the rabbit as early as at embryonic day 25. Only at 3 postnatal weeks, the menisci also contain collagen type II, which increases with further maturation (Bland & Ashhurst, 1996).

The main orientation of collagen fibers is circumferential. Radial fibers are also found but are less numerous. These latter may act as a “tie”, holding the circumferential fibers together and, thereby, resisting longitudinal splitting of the menisci (Bullough et al., 1970; Merkel, 1980; Beaupre et al., 1986; Ghosh & Taylor, 1987).

### Proteoglycans

Less than 1% of the meniscus is constituted by proteoglycans (Fithian et al., 1990). About 80% of the total GAGs in the menisci have been identified as sulfated (Herwig et al., 1984). Normal human meniscal proteoglycans include approximately 40% chondroitin-6-sulphate, 10–20% chondroitin-4-sulphate, 20–30% dermatan-sulphate, and 15% keratan-sulphate (Herwig et al., 1984). These proportions are maintained under tissue culture conditions by a corresponding GAG production (Verbruggen et al., 1996).

Aggrecan has been found the major proteoglycan in human adult menisci (McNicol & Roughley, 1980). Significant regional variations in the meniscal distribution of different GAGs have been reported (Nakano et al., 1997). In dry weight, the inner third of the meniscal body contains 8% GAGs, and its peripheral third only 2%. Biglycan and fibromodulin were found in higher amounts in the inner and middle than the peripheral zones, whereas decorin showed the reverse order (Nakano et al., 1997; Scott et al., 1997). Hyaluronic acid accounted for 4–5% of the total GAG content in the inner third, and for 10% in the peripheral third (Nakano et al., 1997).

### COMP (cartilage oligomeric matrix protein)

**Fig. 2:**
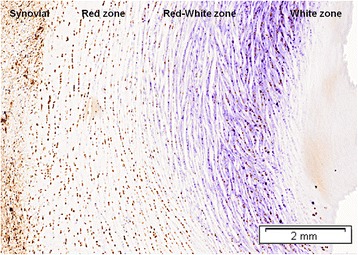
Transversal section of the medial meniscus from a healthy rabbit knee immunostained for COMP. The extracellular matrix revealed the same heterogeneous distribution as for the cells, decreasing from the red to the white zone

The different components of meniscus extracellular matrix interact intensively with each other and with the meniscal cells. One of these macro- molecules is the non-collagenous glycoprotein cartilage oligomeric matrix protein. Cartilage oligomeric matrix protein (COMP) is an integral structural component of the cartilage matrix (Hauser et al., 1995; Rosenberg et al., 1998; DiCesare et al., 1994; Holden et al., 2001) and plays an important role in extracellular matrix assembly (Di Cesare et al., 1999) binding to types I, II and IX collagen, I/II procollagen and chondrocytes (Rosenberg et al., 1998; Holden et al., 2001; Hedbom et al., 1992; Thur et al., 2001). It plays a role in the storage and delivery of hydrophobic hormones (Guo et al., 1998), and it is considered a calcium-binding protein (Thur et al., 2001). High amounts of COMP were detected in the meniscus (Neidhart et al., 1997) and the study of COMP in extracellular matrix revealed the same heterogeneous distribution than cells, decreasing from the red to the white zone (Lopez-Franco et al., 2011) (Fig. 2).

## Meniscus in the degenerative knee

### Gross anatomy (Fig. 1b)

Menisci from OA knees may not preserve their anatomical shape. Usually, medial menisci from OA varus knee show significant changes in the body and the posterior horn (Katsuragawa et al., 2010).

Menisci from patients of advanced age appear yellowish and more opaque, compared to healthy younger menisci. Their surface is usually rough, even without fibrillation (Pauli et al., 2011). OA menisci are dark yellow to brown or reddish color. Calcium deposition on roughened and fibrillated surfaces are often present. Also, tears are frequently observed (Pauli et al., 2011).

### Cell population

Cellular and molecular changes have been reported along joint degeneration, both in menisci from human OA knees and from animal models after experimentally induced OA (Hellio Le Graverand et al., 2001c; Lopez-Franco et al., 2011; Hellio Le Graverand et al., 2001d; Lopez-Franco et al., 2016). A decrease in meniscal cell population complicates the maintenance of a normal meniscus extracellular matrix and, therefore, alters the normal meniscal functions. Nishida et al., 2005 observed that meniscal cell number was markedly decreased 48 weeks after developing a bucket-handle tear trapped in the intercondylar notch. Ochi et al., 1997 noted degenerative changes in menisci after 8 weeks of immobilization, with a decrease of living fibrochondrocytes from 98.7 cells per 0.1 mm^2^ to 28.4. In human OA, López-Franco et al. (Lopez-Franco et al., 2016) observed a statistically significant decrease of meniscal cells compared to non-OA younger menisci. Pauli et al., 2011 also observed decreased cellularity in OA menisci, which was not observed in elderly non-OA menisci.

### Proliferating cells

After injury, proliferating cells may be involved in the maintenance of the meniscus. When the tissue is badly damaged, cells could be unable to replicate themselves in animal experiments. Ochi et al. (Ochi et al., 1997) observed proliferation of fibrochondrocyte-like cells in mature rabbits after removing a long-leg cast. Nishida et al. (Nishida et al., 2005) even observed cells undergoing division. The study of medial menisci from normal and ACL deficient rabbit knees using Ki-67 demonstrated mitotic cells (Lopez-Franco et al., 2011; Hellio Le Graverand et al., 2001b). López-Franco et al. (Lopez-Franco et al., 2016) reported Ki-67 positive cells in all menisci of their control group and only in one of the osteoarthritic group.

### Necrosis and apoptosis

The decrease in cell population during meniscal degeneration is not only related to the limited regenerative ability but also to accidental cell death (necrosis) and programmed cell death (apoptosis). Kwok et al. (Kwok et al., 2016) observed visually prominent cell death (denoted by empty lacuna and pyknotic nuclei) only in menisci from OA murine knees, but not in menisci from normal aging mice. Hashimoto et al. (Hashimoto et al., 1999) suggested that pathological changes in the meniscus are associated with cell apoptosis, and observed that the percentage of apoptotic cells correlated with the severity of tissue destruction. Studying ACL deficient rabbit knees, López-Franco et al. (Lopez-Franco et al., 2011) observed apoptotic cells in 60% (3/5) of the medial menisci 4 weeks after the ACL transection, but only in 20% (1/5) at the contralateral control group. Later, our group (Lopez-Franco et al., 2016) also reported apoptotic cells in 70% of the human osteoarthritic menisci and in only in 20% of the control menisci. This suggests that apoptosis is induced during human meniscal degeneration and could be a putative mechanism in the development of OA.

### Cells clusters (Fig. 3)

Cell clusters have been observed in the meniscus during the early stages (Hellio Le Graverand et al., 2001c; Lopez-Franco et al., 2011; Hellio Le Graverand et al., 2001d; Hellio Le Graverand et al., 2001b; Hashimoto et al., 1999) and in the final stages of OA (Pauli et al., 2011; Katsuragawa et al., 2010; Lopez-Franco et al., 2016). Although their role is unclear, they may appear only after an injury. These cells were associated with frayed edges and tears, and with superficial areas of degeneration (Pauli et al., 2011; Lopez-Franco et al., 2011). The association of abnormal cell clusters and hypertrophic single cells with increased Safranin-O stain indicates phenotypic transition to a chondrocytic appearance. The increase in cell size could represent hypertrophic differentiation (Pauli et al., 2011). Pauli et al. (Pauli et al., 2011) also observed cell aggregates studying human menisci with advancing age, although not resembling the typical cell clusters described in osteoarthritic meniscus degeneration.

**Fig. 3:**
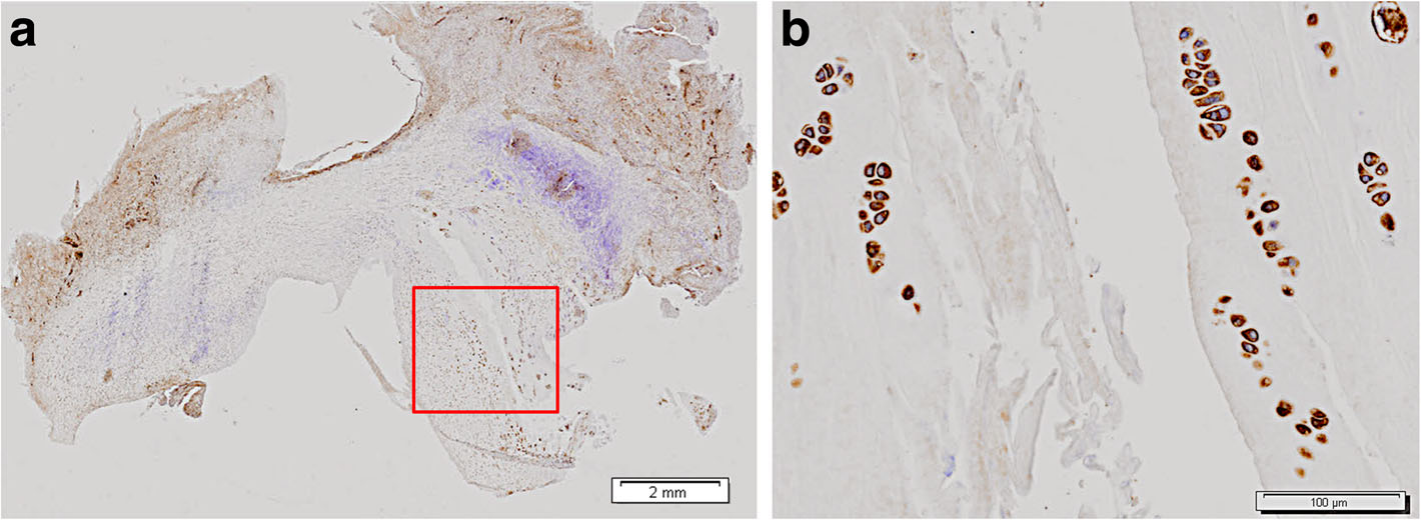
Cell clusters, COMP immunohistochemistry. **a** Microphotograph of a COMP stained medial meniscus 12 weeks after ACL-transection, where a tear can be seen. **b** 100×. Cell clusters, showing chondrocyte-like cells, strongly stain for COMP around the meniscal tear from the boxed area in (**a**)

### Calcification

There is controversy as to whether calcium crystals are a causative factor, a factor that exacerbates the disease, or is simply a bystander in the degenerative disease process of the joint (Sun & Mauerhan, 2012).

Pauli et al. (Pauli et al., 2011) reported that human menisci from osteoarthritic joints showed severe fibrocartilaginous separation of the matrix, tears and calcification. López-Franco et al. (Lopez-Franco et al., 2016) observed deposits of calcium in 16/31 of the menisci from knee joints with diagnosis of idiopathic knee OA that underwent total knee replacement, but none in their control group from young men that suffered an acute meniscal tear. Fuerst et al. (Fuerst et al., 2009) found crystals in 62.5% of meniscal specimens derived from end-stage OA patients. Sun et al. (Sun et al., 2010) examined meniscal specimens derived from end-stage OA patients and found that calcium minerals were present in all meniscal specimens regardless of the patient’s age, but not in any control meniscal specimens. Also, they observed that OA meniscal cells produced more calcium deposits than normal meniscal cells in vitro. Sun and Mauerhan (Sun & Mauerhan, 2012) suggested that meniscal calcification is an early event in the disease process and a predisposing factor for the development of osteoarthritis.

### Collagen

Ghadially et al. (Ghadially et al., 1983) examined human menisci with electron microscopy and observed disruption or fragmentation and parting of collagen fibrils in the injured portions of torn menisci. In the medial meniscus from OA knees, the mean fibril diameter and the percentage of the area occupied by fibrils of collagen were both significantly reduced, and the number of fibrils per area was significantly increased; but in the lateral meniscus, none of these parameters changed significantly with OA (Katsuragawa et al., 2010).

Herwig et al. (Herwig et al., 1984) stated that the content of collagen, expressed as wet weight, decreased in relation to the grade of meniscal degeneration. When referred to the dry weight, they did not observe a consistent correlation. Besides, they reported that alterations in the water, collagen, and GAG content of the menisci in various degrees of degeneration were not due to age-dependent changes.

Hellio Le Graverand et al. (Hellio Le Graverand et al., 2001c) observed that staining of collagen types I and III was increased in both the medial and the lateral meniscus, at 3 and 8 weeks after ACL transection in rabbit knee joints. In contrast, type II collagen staining was overtly increased only in the medial meniscus. At 3 and 8 weeks post-ACL transection, mRNA levels for type I collagen were significantly increased in both the medial and the lateral meniscus. In the medial meniscus, significant increases in mRNA levels for type II collagen were detected at both time periods, while mRNA levels for type III collagen were significantly elevated at 3 weeks. In contrast, mRNA levels for type II collagen were unchanged in the lateral meniscus, and type III collagen mRNA levels were dramatically increased at 3 weeks post-ACL transection (Hellio Le Graverand et al., 2001d).

Katsuragawa et al. (Katsuragawa et al., 2010) studied the rate of collagen neo-synthesis by ^3^H-proline incorporation in OA menisci, and the expression of mRNA for matrix molecules in the menisci. They reported a 9 to 52-fold increase of type I procollagen genes, 3- to 19-fold enhancement of type II procollagen expression, and up to 400-fold increase of type III procollagen. The increase of expression was rather promoted in the medial OA meniscus than in the lateral OA meniscus. In spite of a marked increase in the procollagen gene expression, only modest changes were observed in histology, so an impaired collagen synthesis was suggested.

### Proteoglycans

Adams et al. (Adams et al., 1983) analyzed the GAGs in the dog menisci after sectioning the ACL: after 1 week the GAG content was reduced, reverting to normal only 3–18 months after surgery; the GAGs were elevated above normal levels 15–18 months after surgery. Nishida et al. (Nishida et al., 2005) reported a marked decrease in sulfated GAGs in the dog meniscus at 48 weeks, after undergoing a bucket-handle tear.

Hellio Le Graverand et al. (Hellio Le Graverand et al., 2001c) observed marked degenerative changes at 8 weeks after ACL transection in the extracellular matrix of the medial meniscus, with extensive alteration in the proteoglycan staining pattern, with areas of high intensity and areas of absent staining. Analysis of specific mRNA levels by RT-PCR demonstrated complex changes in both menisci following ACL transection (Hellio Le Graverand et al., 2001d). At 3 and 8 weeks post-ACL transection, mRNA levels for decorin were significantly depressed both in the medial and the lateral meniscus. In the medial meniscus, significant increases in mRNA levels for biglycan were detected at both time periods, while mRNA levels for aggrecan were significantly elevated at 3 weeks post-ACL transection. In contrast, mRNA levels for aggrecan were unchanged in the lateral meniscus, and a significant increase in mRNA levels for biglycan were detected at 8 weeks post-ACL transection (Hellio Le Graverand et al., 2001d). Katsuragawa et al. (Katsuragawa et al., 2010) reported an increase of aggrecan expression in OA menisci, which never exceeded 2-fold.

**Fig. 4:**
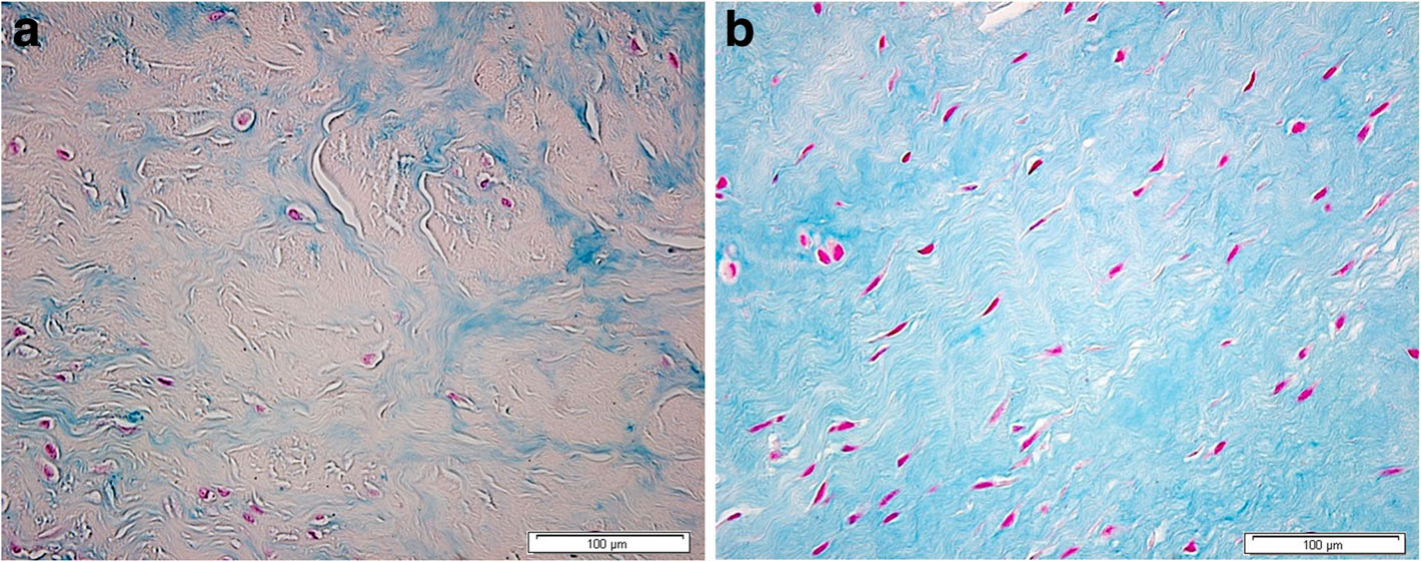
Rabbit meniscus stained with Alcian blue. The extracellular matrix proteoglycans are decreased in (**a**) the OA meniscus, compared with (**b**) the healthy meniscus. Magnification: × 40

Changes in the GAG synthesis and organization can lead to pronounced extracellular framework disturbances. Our group (Lopez-Franco et al., 2016) observed a tendency towards proteoglycan decrease in human OA menisci (Fig. 4), and suggested that these changes could contribute to meniscal degeneration.

## Comp

**Fig. 5:**
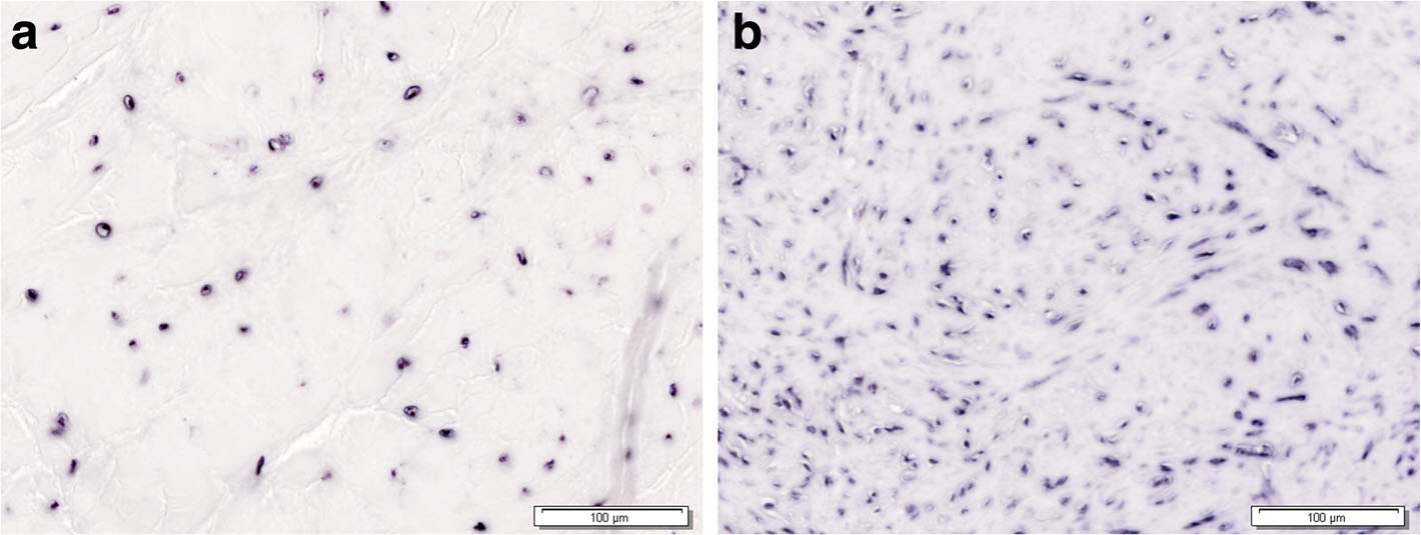
In situ hybridization. Human medial menisci (red zone) stained with a specific riboprobe for human COMP. Cells are decreased in (**a**) the OA meniscus, compared with (**b**) the non OA meniscus

The mean number of COMP-positive cells per high-power field (HPF) in the rabbit medial menisci decreased from 80 ± 25 to 77 ± 13 at 12 weeks after ACL transection (Lopez-Franco et al., 2011). Nevertheless, the study of extracellular matrix in these menisci showed a COMP increase 4 weeks after ACL transection. At 12 weeks, COMP remained elevated, suggesting an increase in cellular activity after aggression in order to protect the tissue. Also, our group (Lopez-Franco et al., 2016) observed a smaller percentage of immunostained matrix and a diminished number of positive nuclei against COMP in menisci from OA knees, compared to samples from partial meniscectomy (Fig. 5). This suggested a significant correspondence between the decrease of COMP and OA development. These data point to a new role for COMP in protecting cells against death: inhibition of apoptosis may be a therapeutic value after cartilage injuries. The potential benefit of COMP in menisci may be hypothesized, but definite proofs are lacking.

## Conclusions

Both experimental animal models of OA and studies on human degenerative knee joint tissues have demonstrated cellular and molecular alterations in menisci. Cell population reduction, extracellular matrix disorganization, and disturbances in collagen and non-collagen protein synthesis and/or expression, are the changes that have been found in the menisci along with knee degeneration. These changes are possibly disease-specific and also different from changes due to aging, although proofs are indirect.

In view of the essential role of menisci in preserving adequate knee function, changes in the menisci may represent a causative factor of the disease or of exacerbation. However, these could just be simple witnesses of OA progression. More basic research is needed to determine causation if animal models incorporating molecular aggression could be followed for degeneration. Also, the relationship of early knee degeneration and early meniscal changes could help to understand the OA progression process. These cannot be studied in the natural disease because the time of OA onset is usually unknown. In contrast, the initial phases of the disease can be studied in the experimentally induced OA with the added advantage that control tissue is available from the same animal, thus eliminating variations between individuals. By these means, other molecular pathways may be revealed as initiators of the degenerative process, whether in the cartilage or in the menisci.

Another area of potential research is related to meniscal repair and knee degeneration. The tissue quality obtained by meniscal repair and meniscus replacement with scaffolds is currently unknown. These techniques, with a wide clinical use, are considered to provide a mechanical benefit for the knee. Clinically, the long-term effect in avoiding or limiting knee degeneration is still unclear. Furthermore, the cellular and biochemical sequence of events in these repaired or reconstructed menisci is unknown, and both descriptive and experimental studies will be required to understand the mechanical and the biological role of these techniques in limiting or delaying knee degeneration. At some point, experiments fostering regeneration of the meniscus structure and composition by cell therapy or other techniques may also enlighten new therapeutic approaches to this complex phenomenon of joint degeneration.

So far, the link of joint degeneration and meniscal changes is well established, but our current knowledge is clearly insufficient to benefit patients with new therapeutic strategies. Further defining cellular and biochemical meniscal changes along knee degeneration in patients, or defining the relationship between chondral damage and meniscal damage are future directions of research that may pave the way to new understanding of knee degeneration and its management.
